# Antibacterial and Antitumor Activities of Biscoumarin and Dihydropyran Derivatives

**DOI:** 10.3390/molecules200917614

**Published:** 2015-09-23

**Authors:** Yun-Peng Sui, Hai-Ru Huo, Jia-Jia Xin, Jing Li, Xiao-Jun Li, Xin-Liang Du, Hai Ma, Hai-Yu Zhou, Hong-Dan Zhan, Zhu-Ju Wang, Chun Li, Feng Sui, Ming-Kai Li

**Affiliations:** 1 Institute of Chinese Materia Medica, China Academy of Chinese Medical Sciences, Beijing 100700, China; E-Mails: suiyp8888@sina.com (Y.-P.S.); hairuhuo@gmail.com (H.-R.H.); hma@icmm.ac.cn (H.M.); zhouhaiyu1992@foxmail.com (H.-Y.Z.); baiyihongdan@163.com (H.-D.Z.); zjwang@icmm.ac.cn (Z.-J.W.); cli@icmm.ac.cn (C.L.); 2 Beijing Chao-Yang Hospital, Capital Medical University, Beijing 100020, China; 3 Department of Blood Transfusion, Xijing Hospital, the Fourth Military Medical University, Xi’an 710032, China; E-Mail: xinjiajia@hotmail.com; 4 The Key Laboratory for Surface Engineering and Remanufacturing in Shaanxi Province, School of Chemical Engineering, Xi’an University, Xi’an 710065, China; E-Mails: lijing3157@aliyun.com (J.L.); xjli@xawl.edu.cn (X.-J.L.); 5 Graduate School of China Academy of Chinese Medical Sciences, Beijing 100700, China; E-Mail: dxl906@163.com; 6 Department of Pharmacology, School of Pharmacy, the Fourth Military Medical University, Xi’an 710032, China

**Keywords:** biscoumarin, dihydropyran, antibacterial, antitumor

## Abstract

A novel series of biscoumarin (**1**–**4**) and dihydropyran (**5**–**13**) derivatives were synthesized via a one-pot multicomponent condensation reaction and evaluated for antibacterial and antitumor activity *in vitro*. The X-ray crystal structure analysis of four representative compounds, **3**, **7**, **9** and **11**, confirmed the structures of these compounds. Compounds **1**–**4** showed the most potent antitumor activity among the total **13** derivatives; especially for compounds **1** and **2**, they also emerged as promising antibacterial members with better antibacterial activity. In addition, the results of density functional theory (DFT) showed that compared with compounds **3** and **4**, biscoumarins **1** and **2** had lower intramolecular hydrogen bonds (HB) energy in their structures.

## 1. Introduction

Natural products have a profound impact on both chemical biology and drug discovery, and the great structural diversity of natural products with various interesting biological characteristics has always provided medicinal chemists an important source of inspiration in their search for new molecular entities with pharmacological activity [1,2]. Among them, biscoumarin and dihydropyran derivatives are two important groups of compounds covering a wide range of biological properties, including anti-oxidant, anti-inflammatory [3] and anti-microbial [4] as well as anticancer activities [5,6]. However, many of them are not suitable for therapeutic application due to their relatively lower activity or evident side-effect properties; and for the already marketable antibacterial and antitumor drugs [7,8], resistance has become one of main reasons for their failure in chemotherapy. Hence the search for high-quality novel antibacterial and anticancer agents has always been advisable and emergent [9].

**Figure 1:**
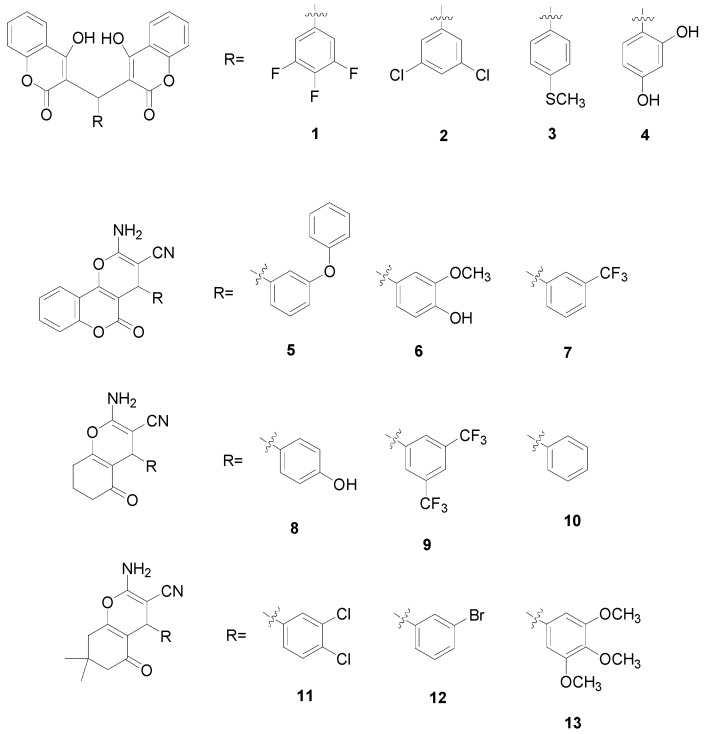
Chemical structures of compounds **1**–**13**.

In order to get more effective antibacterial and antitumor agents, it is possible to make modifications on active chemical structures of title compounds. In the present study, a novel series of biscoumarin (**1**–**4**) and dihydropyran (**5**–**13**) derivatives were firstly synthesized (Figure 1), their antibacterial activities were then measured *in vitro* against *Staphylococcus aureus* (*S. aureus* ATCC 29213), methicillin-resistant *S. aureus* (MRSA XJ 75302), vancomycin-intermediate *S. aureus* (Mu50 ATCC 700699), and USA 300 (Los Angeles County clone, LAC), and finally their antitumor activities on intestinal epithelial adenocarcinoma cell line (HuTu80), mammary adenocarcinoma cell line (4T1) and pancreatic cancer cell line (PANC1) *in vitro* were then evaluated.

## 2. Results and Discussion

### 2.1. Molecular Structure

**Figure 2:**
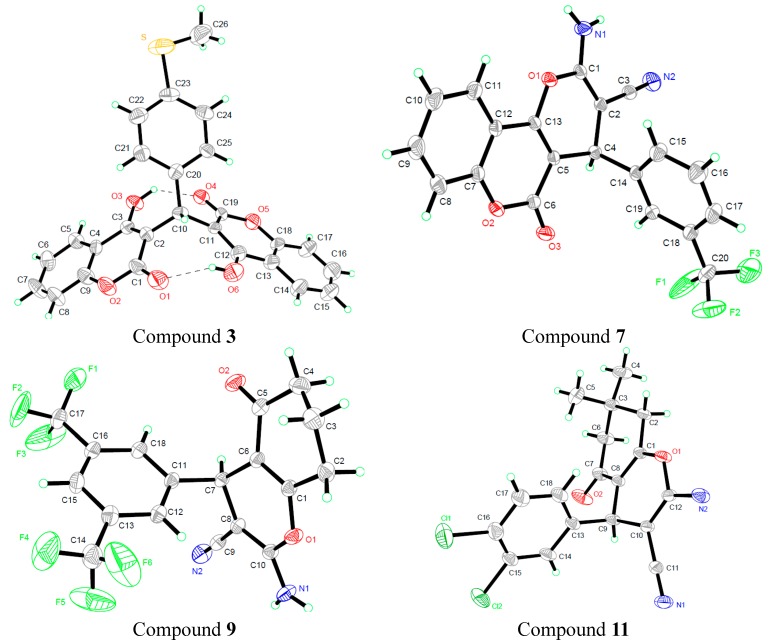
Crystal structures of compounds **3**, **7**, **9** and **11**.

The crystal structures of compounds **3**, **7**, **9** and **11** are given in Figure 2. In the crystal structure of compound **3**, two 4-hydroxycoumarin moieties are linked through a methylene bridge, wherein one hydrogen atom has been replaced with a 4-methylthiophenyl group; and two classical intramolecular hydrogen bonds (O_3_–H_3_···O_4_ and O_6_–H_6_···O_1_) between a hydroxyl group of one coumarin fragment and a lacton carbonyl group of another coumarin fragment further stabilize the whole structure.

In the crystal structures of compounds **7**, **9** and **11**, the new formed pyran ring and the adjacent ketone (coumarin) ring are both basically planar, and the two planes are also essentially parallel to each other. However, the aromatic ring makes a torsion angle to the pyran ring in the three compounds.

### 2.2. Hydrogen Bonds Energies in Biscoumarins ***1**–**4***

We only used compound **1** as an example to estimate single and total intramolecular hydrogen bonds (HB) energies. The global minimum structure is stabilized by two HBs (**1ab**); two higher energy structures are stabilized by one HB (**1a** and **1b**, respectively). The corresponding values are listed in Table 1.

Based on our previous calculation results [10], B3LYP/6-31G* exhibited sufficient agreement with experimental data and lower computational cost, so further theoretical study was performed at this level.

The O_6_–H_6_ O_1_ HB energy was calculated to be −51.3364015 kJ/mol by the equation E(O_6_–H_6_···O_1_) = ${\text{E}}_{\mathrm{lab}}^{\mathrm{coor}}$ – ${\text{E}}_{\mathrm{la}}^{\mathrm{coor}}$, from the energy difference between **1ab** and **1a**, where **1a** is a global minimum structure with O_3_–H_3_···O_4_ HB. Similarly, the O_3_–H_3_···O_4_ HB energy was calculated to be −64.8682285 kJ/mol from the energy difference between **1ab** and **1b** by the equation E(O_3_–H_3_···O_4_) = ${\text{E}}_{\mathrm{lab}}^{\mathrm{coor}}$ − ${\text{E}}_{\mathrm{lb}}^{\mathrm{coor}}$, in which **1b** was obtained from the global minimum structure **1ab**, but H_3_ was rotated around the C_3_–O_3_ bond until O_3_–H_3_···O_4_ HB rupture occurred [11,12]. The total HB energy was calculated to be −116.20463 kJ/mol by the equation E(O_3_–H_3_···O_4_)+ E(O_6_–H_6_···O_1_). For compounds **2**–**4**, the total HB energies are −115.7031595, −118.0346035 and −121.970228 kJ/mol, respectively.

**Table 1:** Single and total intramolecular hydrogen bonds (HB) energies in biscoumarins **1**–**4**.

System	Total Electronic Energies ^a,b^	E(O_6_–H_6_···O_1_)	E(O_3_–H_3_···O_4_)	E(Total HB) ^c^
**1ab**	−1711.038294			−116.20463
**1a**	−1711.018741	−51.3364015		
**1b**	−1711.013587		−64.8682285	
**2ab**	−2332.538245			−115.7031595
**2a**	−2332.518779	−51.107983		
**2b**	−2332.513642		−64.5951765	
**3ab**	−2025.68685			−118.0346035
**3a**	−2025.666896	−52.389227		
**3b**	−2025.661847		−65.6453765	
**4ab**	−1625.791083			−121.970228
**4a**	−1625.770804	−53.2425145		
**4b**	−1625.764906		−68.7277135	

^a^ ZP corrected; ^b^ hartree; ^c^ kJ/mol.

### 2.3. Minimal Inhibitory Concentration (MIC) Assay

For compounds **1**–**13**, one drug-sensitive *S. aureus* (*S. aureus* ATCC 29213) strain and three MRSA strains (MRSA XJ 75302, Mu50, and USA 300 LAC) were used in the systematic analysis of their antibacterial activities *in vitro*. Because of the liposolubility of these compounds, they were dissolved into the solution with 1% dimethyl sulfoxide (DMSO) at final concentration. From Table 2, we can see that, among these compounds, compounds **1** and **2** exerted more potent anti-bacterial activity against the tested *S. aureus* with minimum inhibitory concentration (MIC) values in the range of 2–16 μg/mL. Compared with compounds **1**–**13**, the MIC values of levofloxacin, ceftazidime, ceftriaxone, gentamicin and piperacillin against *S. aureus* (ATCC 29213) strains were lower (less than 8 μg/mL) but were higher against other three strains at varying degrees.

**Table 2:** Minimum inhibitory concentration (MIC) of compounds **1**–**13** and antibiotics in Mueller–Hinton Broth Culture.

Drugs	MIC (µg/mL)			
	*S. aureas* (ATCC 29213)	MRSA (XJ 75302)	Mu50 (ATCC 700699)	LAC (USA 300)
Compound **1**	16	16	8	8
Compound **2**	4	4	2	2
Compound **3**	64	64	64	64
Compound **4**	>256	>256	>256	>256
Compound **5**	>256	>256	>256	>256
Compound **6**	>256	>256	>256	>256
Compound **7**	>256	>256	>256	>256
Compound **8**	>256	>256	>256	>256
Compound **9**	>256	>256	>256	>256
Compound **10**	>256	>256	>256	>256
Compound **11**	>256	>256	>256	>256
Compound **12**	>256	>256	>256	>256
Compound **13**	>256	>256	>256	>256
Levofloxacin	<0.125 (*S*)	4 (*R*)	4 (*R*)	8 (*R*)
Ceftazidime	8 (*S*)	>256 (*R*)	256 (*R*)	64 (*R*)
Ceftriaxone	2 (*S*)	>256 (*R*)	256 (*R*)	32 (*R*)
Gentamicin	0.12 (*S*)	64 (*R*)	32 (*R*)	0.25 (*S*)
Piperacillin	2 (*S*)	>128 (*R*)	>128 (*R*)	8 (*R*)

*S* means drug susceptibility, *R* means drug resistance.

### 2.4. In Vitro Antitumor Activity

Intestinal epithelial adenocarcinoma cell line (HuTu80), mammary adenocarcinoma cell line (4T1) and pancreatic cancer cell line (PANC1) representing three different tumor types were used in the systematic analysis of the antitumor activities of the newly synthesized compounds **1**–**13** *in vitro*. For comparison purpose, the cytotoxicity of carboplatin, a standard antitumor drug, was evaluated under the same condition.

The results showed that all the tested compounds possessed a certain degree of antitumor activities against the three tumor cell lines and their inhibitory action get stronger with the corresponding higher concentration. The related half maximal inhibitory concentration (IC_50_) and IC_90_ values (dose of the compound which cause a 50% and 90% reduction of survival values, respectively) are shown in Table 3. As can be seen in Table 3, there is great difference in the antitumor activity between the four different groups of the tested compounds. Biscoumarins **1**–**4** from the first group showed more potent antitumor activity against the three tested tumor cells (HuTu80, 4T1 and PANC1) with IC_50_ and IC_90_ values of 18.78–32.63 μg/mL and 36.05–64.55 μg/mL, respectively, which is much lower than the IC_50_ and IC_90_ values (45.85–65.62 μg/mL and 102.14–126.24 μg/mL) of the positive control drug carboplatin. However, the compounds in other three groups demonstrated lower antitumor activity with relatively higher IC_50_ and IC_90_ values.

**Table 3:** IC_50_ and IC_90_ values of compounds **1**–**13** and carboplatin against three tumor cell lines (μg/mL).

Drugs	HUTU 80		4T1		PANC1	
	IC_50_	IC_90_	IC_50_	IC_90_	IC_50_	IC_90_
Compound **1**	32.63	64.55	22.09	39.58	27.52	51.33
Compound **2**	28.94	55.87	18.78	36.05	25.05	46.67
Compound **3**	28.42	55.31	20.08	38.21	28.01	51.75
Compound **4**	28.55	55.61	19.33	36.92	26.11	48.56
Compound **5**	116.00	216.00	138.00	248.07	79.23	154.62
Compound **6**	130.67	264.00	163.67	304.96	149.42	302.09
Compound **7**	209.00	409.00	91.44	186.16	174.71	339.00
Compound **8**	266.79	483.83	214.87	330.92	172.99	317.39
Compound **9**	582.88	1079.16	333.93	638.81	744.59	1340.72
Compound **10**	493.58	942.01	303.98	601.82	566.14	1055.13
Compound **11**	268.72	490.94	316.54	569.07	305.25	592.40
Compound **12**	509.78	985.97	621.34	1117.62	604.45	1143.73
Compound **13**	374.50	707.83	481.83	900.82	205.17	410.09
Carboplatin	65.62	126.24	45.85	102.13	52.94	109.94

The IC_50_ (dose of the compound which caused a 50% reduction of survival) and IC_90_ (dose of the compound which caused a 90% reduction of survival) values were calculated from dose-response curves done in triplicate for each compound. Carboplatin was used as positive control.

## 3. Experimental Section

### 3.1. Apparatus and Materials

IR spectra (400–4000 cm^−1^) were obtained using a Brucker Equinox-55 spectrophotometer (Bruker Optics, Ettlingen, Germany). ^1^H-NMR (Nuclear Magnetic Resonance) spectra, ^13^C-NMR spectra and mass spectra were tested using the Varian Inova-400 spectrometer (Varian Inc., Palo Alto, CA, USA), Bruker Avance III (Bruker Optics) spectrometer and micrOTOF-Q II (Bruker Optics) mass spectrometer, respectively. The melting points were taken on a XT-4 micro melting apparatus (Ledon, Suzhou, China), and the thermometer was uncorrected.

All antibiotics used were purchased from the National Institute for the Control of Pharmaceutical and Biological Products (Beijing, China). MRSA (XJ 75302) was isolated from cultures of sputum samples from patients in Xijing Hospital (Xi’an, China). *S. aureus* strain (ATCC 29213) was purchased from the Chinese National Center for Surveillance of Antimicrobial Resistance (Beijing, China). Mu50 (ATCC 700699) and USA 300 (LAC) were purchased from MicroBiologics (Saint Cloud, MN, USA).

RPMI 1640 medium, trypsin, and heat inactivated fetal bovine serum (HIFBS) were obtained from Gibco (New York, NY, USA). 5-Fluorouracil 99% HPLC grade, phosphate-buffered saline (PBS), penicillin/streptomycin (PS) solution, and 3-(4,5-dimethylthiazol-2-yl)-2-5-diphenyltetrazolium bromide (MTT) reagent were purchased from Sigma-Aldrich (St. Louis, MO, USA). The MTT assay was performed by using FlexStation 3 benchtop multi-mode microplate reader (Molecular Devices, San Jose, CA, USA). Human intestinal epithelial adenocarcinoma cell line (HuTu80) was purchased from Institute of Basic Medical Sciences (IBMS) of Chinese Academy of Medical Sciences (CAMS) (Beijing, China). The cells were cultured in RPMI supplemented with 10% HIFBS and 1% PS. Cells were cultured in a 5% CO_2_ in a humidified atmosphere at 37 °C.

### 3.2. Synthesis and Characterization of Compounds ***1**–**13***

Biscoumarins **1**–**4** were synthesized according to the methods of a previous report [13]. A mixture of 3,4,5-trifluorobenzaldehyde (3,5-dichlorobenzaldehyde, 4-methylthiobenzaldehyde or 2,4-dihydroxybenzaldehyde) (10 mmol) and 4-hydroxycoumarin (20 mmol) was dissolved in 100 mL of EtOH. A few drops of piperidine were added, and the mixture was stirred for 3 h at room temperature. After reaction completion as determined by TLC, water was added until precipitation occurred. After filtering the precipitates, they were sequentially washed with ice-cooled water and ethanol and then dried in a vacuum.

*3,3′-(3,4,5-Trifluorobenzylidene)-bis-(4-hydroxycoumarin)* (**1**): Yield: 57%. 230–231 °C. IR (KBr pellet cm^−1^): 3446, 2360, 1675, 1512, 1265, 1139, 767 cm^−1^. ^1^H-NMR (CDCl_3_, δ, ppm): 6.020 (s, 1H, CH), 6.854–6.890 (q, 2H), 7.434–7.464 (q, 4H), 7.669–7.708 (t, 2H), 8.031–8.108 (q, 2H), 11.337 (s, 1H, OH), 11.665 (s, 1H, OH). ^13^C-NMR (DMSO-*d*_6_) δ: 36.462, 103.773, 111.689, 111.729, 111.895, 116.431, 118.642, 124.072, 124.439, 132.355, 152.868, 164.907, 166.325. HRMS (ESI^+^): *m*/*z*: calcd for C_25_H_13_F_3_O_6_: 489.0556 [M + Na]^+^; found: 489.0533.

*3,3′-(3,5-Dichlorobenzylidene)-bis-(4-hydroxycoumarin)* (**2**): Yield: 55%. 239–240 °C. IR (KBr pellet cm^−1^): 3446, 2360, 1666, 1546, 1353, 1091, 761 cm^−1^. ^1^H-NMR (CDCl_3_, δ, ppm): 6.036 (s, 1H, CH), 7.106–7.113 (t, 2H), 7.307–7.309 (d, 1H), 7.432–7.468 (t, 4H), 7.666–7.705 (q, 2H), 8.042–8.114 (m, 2H), 11.314 (s, 1H, OH), 11.630 (s, 1H, OH). ^13^C-NMR (DMSO-*d*_6_) δ: 36.672, 103.422, 116.339, 119.160, 123.933, 124.530, 125.667, 126.059, 132.169, 134.153, 146.825, 152.896, 164.774, 167.058. HRMS (ESI^+^): *m*/*z*: calcd for C_25_H_14_Cl_2_O_6_: 503.0060 [M + Na]^+^; found: 503.0069.

*3,3′-(4-Methylthiobenzylidene)-bis-(4-hydroxycoumarin)* (**3**): Yield: 50%. 232–233 °C. IR (KBr pellet cm^−1^): 2605, 1668, 1614, 1349, 1211, 1100, 1029, 910, 767 cm^−1^. ^1^H-NMR (CDCl_3_, δ, ppm): 2.493 (s, 3H, SCH_3_), 6.073 (s, 1H, CH), 7.149–7.170 (d, 2H), 7.223–7.244 (d, 2H), 7.427–7.447 (d, 4H), 7.634–7.677 (m, 2H), 8.015–8.104 (q, 2H), 11.323 (s, 1H, OH), 11.545 (s, 1H, OH). ^13^C-NMR (DMSO-*d*_6_) δ: 15.408, 36.064, 104.671, 116.528, 118.068, 124.354, 124.373, 126.513, 127.932, 132.557, 135.395, 136.867, 152.635, 165.316, 165.392. HRMS (ESI^+^): *m*/*z*: calcd for C_26_H_18_O_6_S: 481.0716 [M + Na]^+^; found: 481.0779.

*3,3′-(2,4-Dihydroxybenzylidene)-bis-(4-hydroxycoumarin)* (**4**): Yield: 55%. m.p. 262–263 °C. IR (KBr): 3260, 2230, 1681, 1611, 1539, 1392, 1184, 910, 757 cm^−1^. ^1^H-NMR (DMSO-*d*_6_, δ, ppm): 5.635 (s, 1H), 6.568–6.595 (q, 1H), 6.713–6.718 (d, 1H), 6.978–6.999 (d, 1H), 7.318–7.373 (m, 1H), 7.445–7.505 (q, 2H), 7.582–7.624 (m, 1H), 7.683–7.726 (m, 1H), 8.086–8.108 (q, 2H), 9.792 (s, 1H). ^13^C-NMR (DMSO-*d*_6_) δ: 103.214, 114.327, 116.652, 116.936, 123.182, 124.434, 125.018, 129.643, 132.579, 132.912, 152.422, 157.792, 160.985. HRMS (ESI^+^): *m*/*z*: calcd for C_25_H_16_O_8_: 467.0737 [M + Na]^+^; found: 467.0779.

Dihydropyran derivatives (**5**–**13**) were also synthesized according to a reported procedure [14]. A mixture of 4-hydroxycoumarin (3,5-cyclohexanedione, or 1,1-dimethyl-3,5-cyclohexanedione) (10 mmol), aromatic aldehydes (10 mmol), malononitrile (10 mmol) and 4-(dimethylamino)pyridine (DMAP) (1 mmol) in ethanol (100 mL) was refluxed for 2–3 h and then cooled to room temperature. After filtering the precipitates, they were sequentially washed with ice-cooled water and ethanol and then dried under a vacuum.

*2-Amino-4-(3-phenoxyphenyl)-3-cyano-5-oxo-4H,5H-pyrano[3,2c]chromene* (**5**): Yield: 60%. 233–234 °C. IR (KBr pellet cm^−1^): 3688, 2198, 1710, 1670, 1574, 1486, 1374, 1238, 1062, 821 cm^−1^. ^1^H-NMR (DMSO-*d*_6_, δ, ppm): 4.477 (s, 1H, CH), 6.821–6.841 (d, 1H), 6.978–7.041 (m, 4H), 7.112–7.149 (t, 1H), 7.300–7.392 (m, 3H), 7.451–7.519 (m, 4H), 7.707–7.747 (t, 1H), 7.879–7.899 (d, 1H). ^13^C-NMR (DMSO-*d*_6_) δ: 37.241, 58.105, 104.086, 113.430, 117.067, 117.293, 118.560, 119.068, 119.655, 122.981, 123.034, 123.984, 125.159, 130.488, 130.648, 133.463, 146.071, 152.645, 154.038, 156.800, 157.033, 158.476, 160.047. HRMS (ESI^+^): *m*/*z*: calcd for C_25_H_16_N_2_O_4_: 431.1002 [M + Na]^+^; found: 431.1033.

*2-Amino-4-(3-methoxy-4-hydroxyphenyl)-3-cyano-5-oxo-4H,5H-pyrano[3,2c]chromene* (**6**): Yield: 60%. 236–237 °C. IR (KBr pellet cm^−1^): 3415, 3303, 2191, 1686, 1598, 1518, 1374, 1262, 1085, 749 cm^−1^. ^1^H-NMR (DMSO-*d*_6_, δ, ppm): 3.728 (s, 3H, OCH_3_), 4.359 (s, 1H, CH), 6.594–6.619 (q, 1H), 6.690–6.710 (d, 1H), 6.811–6.816 (d, 1H), 7.354 (s, 2H), 7.457–7.511 (q, 2H), 7.690–7.733 (m, 1H), 7.880–7.903 (q, 1H), 8.953 (s, 1H). ^13^C-NMR (DMSO-*d*_6_) δ: 31.181, 36.951, 56.139, 58.781, 104.800, 112.502, 113.532, 115.970, 117.015, 119.889, 120.350, 122.910, 125.097, 133.264, 134.801, 146.201, 147.778, 152.540, 153.516, 158.385, 160.068. HRMS (ESI^+^): *m*/*z*: calcd for C_20_H_14_N_2_O_5_: 385.0795 [M + Na]^+^; found: 385.0770.

*2-Amino-4-(3-trifluoromethylphenyl)-3-cyano-5-oxo-4H,5H-pyrano[3,2c]chromene* (**7**): Yield: 65%. 240–241 °C. IR (KBr pellet cm^−1^): 3408, 3321, 2205, 1701, 1672, 1594, 1381, 1313, 1167, 1109, 1041, 770 cm^−1^. ^1^H-NMR (DMSO-*d*_6_, δ, ppm): 4.654 (s, 1H, CH), 7.467–7.659 (m, 8H), 7.713–7.756 (m, 1H), 7.902–7.926 (q, 1H). ^13^C-NMR (DMSO-*d*_6_) δ: 31.169, 37.242, 57.707, 103.550, 113.473, 117.079, 119.517, 123.065, 124.465, 124.502, 124.841, 124.879, 125.159, 126.020, 129.319, 129.632, 130.165, 132.477, 133.525, 145.164, 152.718, 154.304, 158.506, 160.087. HRMS (ESI^+^): *m*/*z*: calcd for C_20_H_11_F_3_N_2_O_3_: 407.0614 [M + Na]^+^; found: 407.0480.

*2-Amino-4-(4-hydroxyphenyl)-5-oxo-5,6,7,8-tetrahydro-4H-chromene-3-carbonitrile* (**8**): Yield: 60%. 238–239 °C. IR (KBr pellet cm^−1^): 3198, 2200, 1647, 1512, 1367, 1215, 1009, 836 cm^−1^. ^1^H-NMR (DMSO-*d*_6_, δ, ppm): 1.854–1.973 (m, 2H), 2.214–2.302 (m, 2H), 2.573–2.606 (q, 2H), 4.074 (s, 1H, CH), 6.644–6.665 (q, 2H), 6.926–6.947 (q, 4H), 9.263 (s, 1H). ^13^C-NMR (DMSO-*d*_6_) δ: 20.307, 26.931, 31.184, 35.032, 36.860, 59.101, 114.735, 115.464, 120.442, 128.612, 135.710, 156.465, 158.846, 164.475, 196.359. HRMS (ESI^+^): *m*/*z*: calcd for C_16_H_14_N_2_O_3_: 305.0897 [M + Na]^+^; found: 305.0980.

*2-Amino-4-(3,5-ditrifluoromethylphenyl)-5-oxo-5,6,7,8-tetrahydro-4H-chromene-3-carbonitrile* (**9**): Yield: 69%. 209–210 °C. IR (KBr pellet cm^−1^): 3397, 2194, 1683, 1651, 1419, 1362, 1330, 1211, 700 cm^−1^. ^1^H-NMR (DMSO-*d*_6_, δ, ppm): 1.889−1.960 (m, 2H), 2.258–2.318 (m, 2H), 2.607–2.709 (m, 2H), 4.543 (s, 1H, CH), 7.239 (s, 2H), 7.857 (s, 2H), 7.968 (s, 1H). ^13^C-NMR (DMSO-*d*_6_) δ: 20.183, 27.055, 31.096, 35.898, 36.679, 57.089, 112.623, 119.735, 121.012, 121.049, 121.082, 122.434, 125.145, 128.644, 130.201, 130.527, 130.851, 131.176, 148.674, 159.034, 166.000, 196.552. HRMS (ESI^+^): *m*/*z*: calcd for C_18_H_12_F_6_N_2_O_2_: 425.0695 [M + Na]^+^; found: 425.0663.

*2-Amino-5-oxo-4-phenyl-5,6,7,8-tetrahydro-4H-chromene-3-carbonitrile* (**10**): Yield: 65%. 221–222 °C. IR (KBr pellet cm^−1^): 3309, 3170, 2194, 1685, 1261, 1064, 999, 702 cm^−1^. IR (KBr pellet cm^–1^): 3309, 3170, 2194, 1685, 1261, 1064, 999, 702 cm^–1^. ^1^H-NMR (DMSO-*d*_6_, δ, ppm): 1.876−1.988 (m, 2H), 2.2262.318 (m, 2H), 2.599–2.640 (q, 2H), 4.183 (s, 1H, CH), 7.019 (s, 2H), 7.146–7.205 (m, 3H), 7.269–7.306 (t, 2H). ^13^C-NMR (DMSO-*d*_6_) δ: 20.295, 26.955, 31.186, 35.922, 36.808, 58.652, 114.255, 120.289, 127.015, 127.609, 128.822, 145.278, 158.948, 164.971, 196.348. HRMS (ESI^+^): *m*/*z*: calcd for C_16_H_14_N_2_O_2_: 289.0947 [M + Na]^+^; found: 289.0933.

*2-Amino-4-(3,4-dichlorophenyl)-3-cyano-7,7-dimethyl-5-oxo-4H-5,6,7,8-tetrahydrobenzo[b]pyran* (**11**): Yield: 66%. 232–233 °C. IR (KBr pellet cm^−1^): 3325, 2958, 2194, 1639, 1608, 1465, 1357, 1249, 1215, 1033, 898 cm^−1^. ^1^H-NMR (DMSO-*d*_6_, δ, ppm): 0.965 (s, 3H, CH_3_), 1.039 (s, 3H, CH_3_), 2.114–2.155 (d, 1H), 2.234–2.274 (d, 1H), 2.529 (s, 2H), 4.255 (s, 1H, CH), 7.137–7.172 (m, 3H), 7.390–7.395 (d, 1H), 7.756–7.582 (d, 1H). ^13^C-NMR (DMSO-*d*_6_) δ: 27.412, 28.689, 31.183, 32.323, 35.439, 50.387, 57.649, 112.193, 119.902, 128.170, 129.652, 129.773, 131.118, 131.286, 146.380, 158.994, 163.461, 196.238. HRMS (ESI^+^): *m*/*z*: calcd for C_18_H_16_Cl_2_N_2_O_2_: 385.0481 [M + Na]^+^; found: 385.0422.

*2-Amino-4-(3-bromophenyl)-3-cyano-7,7-dimethyl-5-oxo-4H-5,6,7,8-tetrahydrobenzo[b]pyran* (**12**): Yield: 68%. 239–240 °C. IR (KBr pellet cm^−1^): 3344, 3168, 2963, 2191, 1686, 1651, 1605, 1469, 1427, 1367, 1250, 1216, 1036, 695 cm^−1^. ^1^H-NMR (DMSO-*d*_6_, δ, ppm): 0.967 (s, 3H, CH_3_), 1.043 (s, 3H, CH_3_), 2.111–2.151 (d, 1H), 2.242–2.82 (d, 1H), 2.534 (s, 2H), 4.210 (s, 1H, CH), 7.096 (s, 2H), 7.154–7.174 (d, 1H), 7.2567.312 (m, 2H), 7.385–7.409 (m. 1H). ^13^C-NMR (DMSO-*d*_6_) δ: 27.272, 28.820, 31.195, 32.337, 35.816, 50.398, 58.091, 112.565, 120.011, 122.047, 126.858, 130.012, 130.417, 131.122, 147.967, 159.002, 163.338, 196.187. HRMS (ESI^+^): *m*/*z*: calcd for C_18_H_17_BrN_2_O_2_: 395.0366 [M + Na]^+^; found: 395.0450.

*2-Amino-4-(3,4,5-trimethoxyphenyl)-3-cyano-7,7-dimethyl-5-oxo-4H-5,6,7,8-tetrahydrobenzo[b]pyran* (**13**): Yield: 60%. 184−185 °C. IR (KBr pellet cm^−1^): 3313, 3186, 2961, 2189, 1656, 1588, 1505, 1459, 1420, 1367, 1322, 1212, 1124, 536 cm^−1^. ^1^H-NMR (DMSO-*d*_6_, δ, ppm): 1.029 (s, 3H, CH_3_), 1.060 (s, 3H, CH_3_), 2.091 (s, 2H), 2.131–2.172 (d, 1H), 2.271–2.311 (d, 1H), 3.633 (s, 3H), 3.727 (s, 6H), 4.145 (s, 1H, CH), 6.389 (s, 2H), 6.975 (s, 2H). ^13^C-NMR (DMSO-*d*_6_) δ: 27.039, 29.098, 31.179, 32.234, 36.098, 50.456, 56.247, 58.802, 60.398, 104.590, 112.816, 120.232, 136.569, 140.966, 153.232, 158.828, 163.272, 196.219. HRMS (ESI^+^): *m*/*z*: calcd for C_21_H_24_N_2_O_5_: 407.1577 [M + Na]^+^; found: 407.1565.

### 3.3. X-ray Crystallography

For X-ray diffraction experiments, single crystals of compounds **3**, **7**, **9** and **11** were grown from methanol. The X-ray diffraction data were collected on a Bruker SMART APEX II CCD diffractometer (Bruker Optics, Ettlingen, Germany) equipped with a graphite monochromated Mo Kα radiation (λ = 0.71073 Å) by using the ω-2θ scan technique at room temperature. The structure was solved by direct methods using SHELXS-97 (Sheldrick 1997, University of Gottingen, Germany) and refined using the full-matrix least squares method on *F*^2^ with anisotropic thermal parameters for all non-hydrogen atoms by using SHELXL-97 [15]. Hydrogen atoms were generated geometrically. The crystal data and details concerning data collection and structure refinement are given in Table 4. Molecular illustrations were prepared using the XP package. Parameters in CIF format are available as Electronic Supplementary Publication from Cambridge Crystallographic Data Centre.

CCDC 1048441-1048444 for compounds **3**, **7**, **9** and **11** contains the supplementary crystallographic data for this paper. These data can be obtained free of charge via http://www.ccdc.cam.ac.uk/conts/retrieving.html (or from the CCDC, 12 Union Road, Cambridge CB2 1EZ, UK; Fax: +44-1223-336033; E-mail: deposit@ccdc.cam.ac.uk) 

**Table 4:** Crystal data, data collection and structure refinement of compounds **3**, **7**, **9** and **11**.

	Compound 3	Compound 7	Compound 9	Compound 11
Formula	C_26_H_18_O_6_S	C_20_H_11_F_3_N_2_O_3_	C_18_H_12_F_6_N_2_O_2_	C_18_H_16_Cl_2_N_2_O_2_
Mr	458.08	384.31	402.3	362.06
Crystal system	Triclinic	Triclinic	Triclinic	Triclinic
Space group	*P*ī	*P*ī	*P*ī	*P*ī
a/Å	10.947 (3)	7.9416 (6)	8.5762 (6)	8.2045 (5)
b/Å	20.402 (7)	10.9917 (9)	8.8891 (8)	14.1658 (8)
c/Å	20.694 (7)	11.4755 (6)	12.1026 (8)	23.3891 (15)
α/°	109.44 (3)	112.429 (7)	109.583 (7)	94.540 (5)
β/°	90.09 (3)	105.094 (6)	92.163 (6)	97.474 (5)
γ/°	93.29 (3)	97.364 (6)	97.257 (7)	98.928 (5)
V/Å^3^	4350 (3)	864.45 (11)	859.13 (11)	2648.9 (3)
*Z*	2	2	2	2
D_calc_/g·cm^−3^	1.401	1.447	1.555	1.357
μ(Mo Kα)/mm^−1^	0.191	0.122	0.146	0.38
θ range/°	2.43 to 25.00	2.74 to 27.68	2.43 to 27.35	2.76 to 27.51
Reflections collected	18355	5790	5819	18090
No. unique data [*R*(int)]	13442 [0.0377]	3045 [0.0233]	3030 [0.0213]	9317 [0.0442]
No. data with *I* ≥ 2σ(*I*)	5431	2170	2318	6552
*R*_1_	0.0783	0.0595	0.0745	0.0824
ω*R*_2_(all data)	0.2469	0.1774	0.2276	0.261

### 3.4. Quantum Chemical Calculations

All calculations were carried out using the Gaussian 09 package [16]. Density functional theory (DFT), Becke’s three-parameter hybrid function (B3LYP), and LYP correlation function were used to fully optimize all the geometries on the energy surface without constraints. To obtain precise results that are in conjunction with experimental results, three basis sets, namely 6-31G*, 6-31 + G**, and 6-311G*, were tested. Frequency calculations at the B3LYP (with basis sets 6-31G*) level of theory were carried out to confirm stationary points as minima and to obtain the zero-point energies and the thermal correlation data at 1 atm and 298 K.

### 3.5. Minimal Inhibitory Concentration (MIC) Assay

Based on the CLSI broth microdilution method [17], the determination of minimum inhibitory concentrations (MICs) via microdilution assay was performed in sterilized 96-well polypropylene microtiter plates (Sigma-Aldrich) in a final volume of 200 μL. Bacteria were grown overnight in nutrient broth. Mueller–Hinton (MH) broth (100 μL) containing bacteria (5 × 10^5^ CFU/mL) was added to 100 μL of the culture medium containing the test compound (0.12 μg/mL–256 μg/mL in serial 2-fold dilutions). The plates were incubated at 37 °C for 20 h in an incubator. About 50 µL of 0.2% triphenyl tetrazolium chloride (TTC), a colorimetric indicator, was added to each well of microtiter plates and incubated at 35 °C for 1.5 h. The TTC-based MIC was determined as the lowest concentration of oxacillin that showed no red color change indicating complete growth inhibition.

### 3.6. Cell Viability Assay

Viability of intestinal epithelial adenocarcinoma cell line (HuTu80), mammary adenocarcinoma cell line (4T1) and pancreatic cancer cell line (PANC1) was determined by using the MTT assay as described by Mosmann [18]. Cells reaching 70%–80% confluency were treated with various concentrations of the synthesized compounds with 1% dimethyl sulfoxide (DMSO) as a negative control. After 48 h incubation, 20 μL of MTT solution (5 mg/mL in PBS) was added and incubated for an additional 4 h. Subsequently, the medium was aspirated carefully, and 150 μL of DMSO was added. After incubation for 15 min, the optical density was measured at 490 nm using FlexStation 3 benchtop multi-mode microplate reader (Molecular Devices). Data were recorded and analyzed for the assessment of the effects of the test substances on cell viability and growth inhibition. The IC_50_ and IC_90_ values were calculated using regression equation as explained before. The results are presented as the average percentage viability to the negative control (1% DMSO). The percentage of cell viability was calculated using the following formula: % cell viability = (absorbance of treated/absorbance of untreated) × 100. The percentage of inhibition was plotted against the concentration in Microsoft excel and the IC_50_ was calculated using the regression equation.

## 4. Conclusions

In this work, we synthesized two new series of biscoumarin and dihydropyran derivatives; evaluated their antibacterial activities *in vitro* against one-drug-sensitive *S. aureus* (*S. aureus* ATCC 29213) strain and three MRSA strains (MRSA XJ 75302, Mu50, and USA 300 LAC); and then measured their antitumor activities on intestinal epithelial adenocarcinoma cell line (HuTu80), mammary adenocarcinoma cell line (4T1) and pancreatic cancer cell line (PANC1) *in vitro*.

In addition, X-ray structural analysis showed that biscoumarins **1**–**4** had two classical intramolecular O–H···O hydrogen bonds in their structures. Their corresponding intramolecular hydrogen bonds energy was calculated to be −116.20463, −115.7031595, −118.0346035 and −121.970228 kJ/mol, respectively.

Among the synthesized compounds, compounds **1**–**4** had more potent antitumor activity against the tested three cancer cell lines with the IC_50_ and IC_90_ values of 18.78–32.63 μg/mL and 36.05–64.55 μg/mL, respectively, which is much lower than that of the positive control drug carboplatin; compounds **1** and **2**, with lower intramolecular hydrogen bonds energy, also showed the most potent antibacterial effect on four *S. aureus* bacterial strains with the MIC values of 2–16 μg/mL. The reason may be that intramolecular HB strength is related to the stability of chemical structure, which further affects the binding affinity between molecules and target protein.
